# Gel Properties and Protein Structures of Minced Pork Prepared with κ-Carrageenan and Non-Meat Proteins

**DOI:** 10.3390/gels10050305

**Published:** 2024-04-30

**Authors:** Yang Ye, Fei Chen, Meimei Shi, Yang Wang, Xia Xiao, Chunmei Wu

**Affiliations:** 1 School of Biological Engineering, Sichuan University of Science and Engineering, Yibin 644005, China; yeyang161@suse.edu.cn (Y.Y.); cf192118@163.com (F.C.); 15708286770@163.com (X.X.); wchm_84@163.com (C.W.); 2 Food Fermentation Industry Research and Design Institute of Sichuan Province, Chengdu 610000, China; 15881657280@163.com

**Keywords:** minced pork, κ-carrageenan, non-meat proteins, gel properties, protein structures

## Abstract

Problems with minced pork include water release and low gel strength. This study aimed to investigate the effect of treatments with κ-carrageenan (κ-CAR), egg white powder (EWP), wheat gluten (WG), soy isolate protein (SPI), and a combination of these treatments on the gel properties and protein structures of minced pork. The cooking loss and trapped water within minced pork increased when additives were incorporated; in particular, the SPI group reached 1.31 ± 0.01% and 91.42 ± 0.20%. The hardness and chewiness of minced pork reached their maximum values (38.91 ± 0.80 N, 14.73 ± 0.41 N) when the WG was added. The κ-CAR/WG-minced pork gel network structure was the densest and most stable, characterized by increased hydrophobic interactions, disulfide bonds in the mince gel, and enthalpy value. The α-helix content with κ-CAR/WG treatment decreased from 27% to 7.8%, transforming into other secondary structures. This suggests that the addition of κ-CAR/WG can be a more effective combination for improving the quality of minced pork.

## 1. Introduction

Pork stands as the most consumed meat in China, making up over 33% of the global meat consumption and approximately half of worldwide consumption and production [1]. Its popularity can be attributed to its richness in protein, fat, and amino acids, minerals, and various trace elements [2]. It is frequently processed and transformed into multiple products, one of which is minced pork. Typically, the process entails dicing the pork and incorporating salt and other excipients to create minced mixture. This is then shaped into balls of varying sizes, and subsequently heated it to produce minced pork gel. The irreversible denaturation of minced pork proteins following heat treatment leads to diminished water-holding capacity (WHC), affecting the chewiness and texture of minced pork [3]. Similar edible gels include those made from silver carp surimi gel [4], chicken meat paste gel [5], and minced beef meat paste gel [6]. Proteins and polysaccharides are the primary materials utilized in food processing to modify the characteristics of various foodstuffs. These include hydrocolloids, non-meat proteins, and soluble dietary fibers [7]. They act synergistically to construct natural phases of the colloidal system, and their interactions affect the key properties of food, such as WHC and thermal stability [8].

κ-CAR (κ-carrageenan) is a naturally occurring linear sulphated polysaccharide extracted from red edible seaweed. The inclusion of 0.3% CAR and 0.7% alginate has been shown to enhance the cooking rate and texture characteristics of duck ham [9]. Additionally, the introduction of 1% CAR markedly boosts the gel strength and viscoelasticity of shrimp myofibrillar fiber gels. However, it is important to note that an abundance of κ-CAR inversely affect gel characteristics, leading to diminished gel quality [10]. Non-meat proteins such as egg white powder (EWP), wheat gluten (WG), and soy isolate protein (SPI) have been utilized as additives. They significantly enhance the gel strength and WHC of myofibrillar proteins (MPs). This improvement is attributed to their gelation properties and their exceptional ability to create an advanced viscoelastic network [11,12,13]. Jin et al. demonstrated that incorporating 1.5% SPI and EWP, respectively, into sausages reduced the cooking loss by more than 6% in comparison to the control group [14]. Similarly, Wang et al. found that pork meatballs modified with WG exhibited a significant reduction in cooking loss [15], which decreased by 49.16% compared to meatballs without added extenders after a 30 min treatment. However, it has been noted in certain studies that excessive amounts of CAR and natural SPI can adversely affect the gelation properties of meat proteins [16,17,18]. Therefore, extensive studies have been carried out on the combination of polysaccharide and protein mixtures to improve the quality of sausages, minced fish, and meat patties. Sun et al. [19] demonstrated that κ-CAR significantly bolstered the gelation properties and water-holding capacity of MP-SPI composite gels. Additionally, meat patties subjected to treatment with the SPI/CAR mixture showed a notable increase in hardness and chewiness [20]. In research conducted by Wasinnitiwong et al. [21], it was found that surimi containing salted duck egg white powder and κ-CAR exhibited superior gelling properties and a more compact microstructure compared to κ-CAR alone. However, the application of polysaccharides and proteins to enhance the gel properties and protein structure of minced pork is underutilized.

Our study aimed to explore the gel properties and protein structures of minced pork treated with κ-CAR, EWP, WG, and SPI individually, as well as combinations of κ-CAR/EWP, κ-CAR/WG, and κ-CAR/SPI. The goal was to enhance the quality of minced pork products through these treatments.

## 2. Results and Discussion

### 2.1. Cooking Loss and WHC

Cooking loss serves as a crucial indicator in the meat processing, signifying the retention of meat juice during heating and ripening. According to the data presented in Table 1, the application of additives significantly decreased cooking losses in comparison to the control group, indicating a more effective retention of juices within the minced pork. The cooking loss observed in minced pork treated with SPI and the combination of κ-CAR/SPI was lower than that in other treatments, indicating that SPI is capable of forming superior gels which exhibit impressive good water and oil retention capacity [22]. These findings align with those reported by Kang, Chen, and Ma [23], who noted a substantial improvement in the cooking yield of frankfurters enhanced with SPI (*p* < 0.05). Shin et al. [24] discovered that incorporating κ-CAR into frankfurters led to elevated cooking yields and textural properties, such as hardness, chewiness, and gumminess. Adding κ-CAR/SPI into the protein matrix has the potential to transform the configuration of meat proteins. This transformation is key to minimizing water loss during cooking [25].

Water-holding capacity (WHC) is commonly employed as an indicator of meat quality and yield [26]. The introduction of additives significantly enhanced WHC when compared to the control group; notably, minced pork treated with SPI demonstrated a superior water-holding capacity, exhibiting an increase of 16.19% over the control. Consistent with the findings of this study, Moirangthem et al. [27] also observed an increase in moisture content with SPI treatment in low-fat duck meat sausages. This enhancement in WHC was attributed to the ability of SPI to absorb and retain substantial qualities of water within the 3D MP network [28]. At this juncture, the formation of an SPI-MP gel physically traps the water, thereby increasing WHC. We hypothesized that the observed effects were due to interactions between CAR, SPI, and the meat proteins. Contrary to the outcomes noted with SPI treatment results, the proteins present in WG absorb water and swell during the gelling process, thus increasing the WHC of the minced pork [29].

### 2.2. Color

Color is predominantly influenced by the protein composition, protein stock, and optical properties of the particles [30]. As displayed in Table 1, there was no marked difference in the L*, a*, and b* values between the control group and mixed treatments of minced meat (*p* < 0.05). Minced pork with additives exhibited lower L* values and higher a* and b* values compared to the control group. In particular, minced pork enhanced with SPI showed lower L* values than those subjected to other treatments. The reduction in L* values in minced pork can be attributed to the light yellow color of the additives. Furthermore, the additives increased the network structure or density of the minced pork, leading to an increase in light reflectance, thus making the color lighter [31]. Zhao et al. also discovered that incorporating CAR into low-salt chicken products results in the diminished whiteness of the final product [5]. The minced pork treated with SPI exhibited a higher water content, which led to a looser gel network structure. This change in gel structure resulted in lower light reflectivity, consequently diminishing the brightness of the product.

### 2.3. Texture Profile Analysis

Texture profile analysis containing four primary parameters (hardness, cohesiveness, springiness, and chewiness) is in Table 2. The addition of EWP, WG, κ-CAR/EWP, and κ-CAR/WG mixture was found to enhance hardness, cohesiveness, springiness, and chewiness compared to the control. The findings relating to the treatment using EWP and the κ-CAR/EWP mixture were consistent with the research presented by Quan and Benjakul, and Tang et al. These studies indicated that gels supplemented with κ-CAR and mixed κ-CAR/SPI demonstrated a more compact network structure, thereby increasing TPA parameters [32,33]. Minced pork supplemented with WG exhibited the best characteristics in terms of hardness, springiness, cohesiveness, and chewiness. This is supported by the study conducted by Chiang et al., which demonstrated an elevation in the hardness and chewiness of the meat analogs proportional to the increased levels of WG [34]. The underlying mechanism involves gliadin’s ability to bind glutenin through non-covalent interactions, such as hydrogen bonding and hydrophobic and ionic interactions [35]. These interactions bestow viscoelastic properties upon the gluten powder, resulting in a denser gel network within the minced meat, thus improving the hardness and elasticity of minced pork. TPA parameters observed in minced pork treated with κ-CAR and SPI displayed a contrary trend compared to the findings of Jiang et al. and Zhang et al. [36,37], who noted that κ-CAR and SPI typically enhance the firmness and chewiness of meat products. According to the investigation by Cao et al. [16], κ-CAR addition was shown to significantly affect the TPA parameters of sausages (*p* < 0.05). From these observations, it can be concluded that the specific composition, quantity, and method by which CAR and SPI are integrated can significantly influence the quality of minced meat.

### 2.4. Protein Secondary Structure

The secondary structure is crucial for the architecture and folding proteins [38]. The Raman spectroscopy analysis of protein secondary structure primarily benefits from the carbonyl stretching mode observed in amide I and the combination of in-plane N-H bending and C-N stretching motion characteristics of amide III. These Raman amide bands serve as pivotal markers and are often used as indicators of protein secondary structure [39]. Each sub-peak corresponds to the secondary structure: α-helix manifests within the range of 1650 to 1660 cm^−1^, β-fold appears from 1665 to 1680 cm^−1^, β-turn is identified at 1680 cm^−1^, and the irregular curl around 1660–1665 cm^−1^.

**Figure 1:**
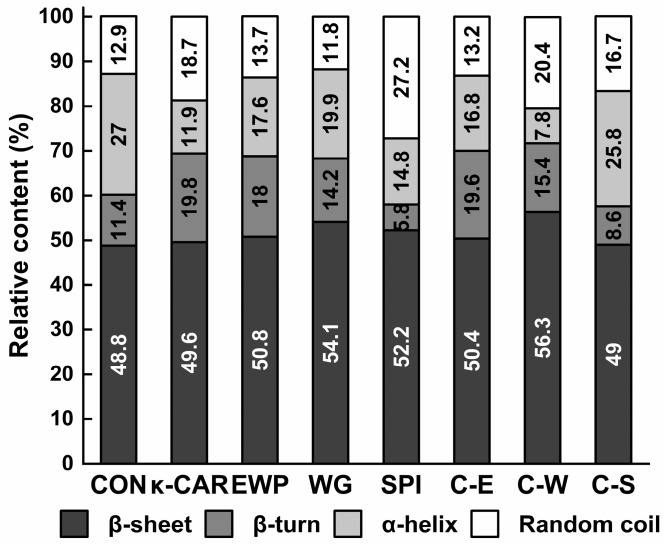
Secondary structure of pork meatballs formulated with different additives. CON, without κ-CAR or proteins; C, κ-CAR (0.5%κ-carrageenan); E, EWP (4%egg white powder); W, WG (6%wheat gluten); S, SPI (5%soy isolate protein).

The secondary structure of minced pork protein gels, depicted through variations in the relative content of α-helix, β-sheet, β-turn, and random coils, is illustrated in Figure 1. The additives were found to markedly reduce the relative content of α-helix while enhancing the total relative content of β-sheet, β-turn, and random coils in comparison to the control. Minced pork proteins formulated with κ-CAR/WG exhibited the most substantial increase in the combined relative content of β-sheet, β-turn, and random coils at 92.1%. The relative content of α-helix dramatically declined from 27% to 7.8%, while that of random coils rose from 12.9% to 20.4%.

During the thermal induction of protein gelation, the unfolding of α-helix structures, and the release of intermolecular hydrophobic residues, which transforms into β-sheet, β-turn, and random coil structures, these structures are positively correlated with hardness [20,40]. Based on our findings, we deduced that the enhanced hardness of pork meatballs containing κ-CAR/WG is associated with the conversion of α-helix into other structures. The increased proportion of random coils in the κ-CAR/WG group correlates with inferior cooking loss and WHC when compared to the κ-CAR/SPI group. Ultimately, these insights underscore the intimate relationship between the secondary structure of minced pork and its water-holding and textural properties.

### 2.5. DSC

In this study, DSC analysis was utilized to investigate the thermal properties of minced pork. DSC analysis provides insight into the stability and internal structure of proteins during denaturation by measuring parameters such as denaturation temperature (Td) and enthalpy (∆H) [41,42]. From Table 3, the addition of κ-CAR, EWP, WG, and SPI had differential impacts on the Td and ∆H of the proteins of the minced pork system to varying degrees. Notably, these additives led to a decrease in denaturation temperature and facilitated an increase in enthalpy compared to the control group. During thermal reactions, enhanced protein distribution and the diminished thickness of the interfacial protein film may lead to a reduction in thermal stability, as well as an increase in ∆H [29]. Relative to other groups, the combinations of κ-CAR/EWP and κ-CAR/WG resulted in elevated denaturation temperatures, reaching 65.4 °C for both, and augmented the ∆H value to 0.51 J/g and 0.75 J/g, respectively. This suggests a decreased probability of protein denaturation and an enhancement in protein conformation, as well as the chemical forces that maintain its stability.

### 2.6. Analysis of Chemical Interactions

**Figure 2:**
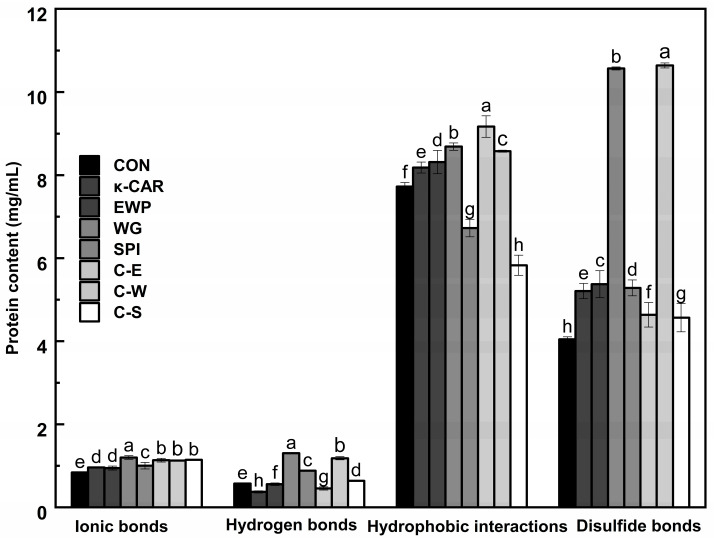
Chemical interaction forces of pork meatball protein formulated with different additives. The error bars indicate SD (n = 3). CON, without κ-CAR and proteins; C, κ-CAR (0.5%κ-carrageenan); E, EWP (4%egg white powder); W, WG (6%wheat gluten); S, SPI (5%soy isolate protein). The different letters indicate significant differences (*p* < 0.05).

The formation of thermally induced gel from minced pork is significantly influenced by various bonding interactions, including ionic bonds, hydrophobic interactions, hydrogen bonds, and disulfide bonds, which are critical in determining the gel properties of proteins [29]. Figure 2 illustrates that the protein gel network structure is primarily composed of hydrophobic interactions and other covalent bonds. These hydrophobic interactions play a pivotal role in the unfolding process of the protein [43]. The reduction in hydrophobic interactions in the SPI and κ-CAR/SPI groups leads to a disruption of the 3D network structure of the gel, aligning with the previously mentioned results of increased irregular curl content [40]. Furthermore, the inclusion of exogenous additives increased the content of disulfide bonds in minced pork compared to the control group, especially in the WG and κ-CAR/WG groups. The high-temperature environment induces the opening of disulfide bonds within the chains of gliadin in gluten flour, enhancing the likelihood of disulfide bond formation between the chains of the gluten network system [44]. The increase in disulfide bonds encourages the formation of a more orderly gel network structure, thereby increasing gel strength [45]. Therefore, it is conjectured that the superior textural characteristics of minced pork in the WG and CAR/WG groups can be attributed to the aforementioned phenomenon. The results showed that the regular transition from of the α-helix to β-sheet structure pattern led to changes in hydrogen bonding and network structure [4]. Hydrogen bonds and ionic bonds account for a relatively small proportion of chemical forces. The minimal presence of ionic bonds across all samples suggests that additives exert an insignificant impact on the ionic bonding within the system.

### 2.7. Scanning Electron Microscopy

**Figure 3:**
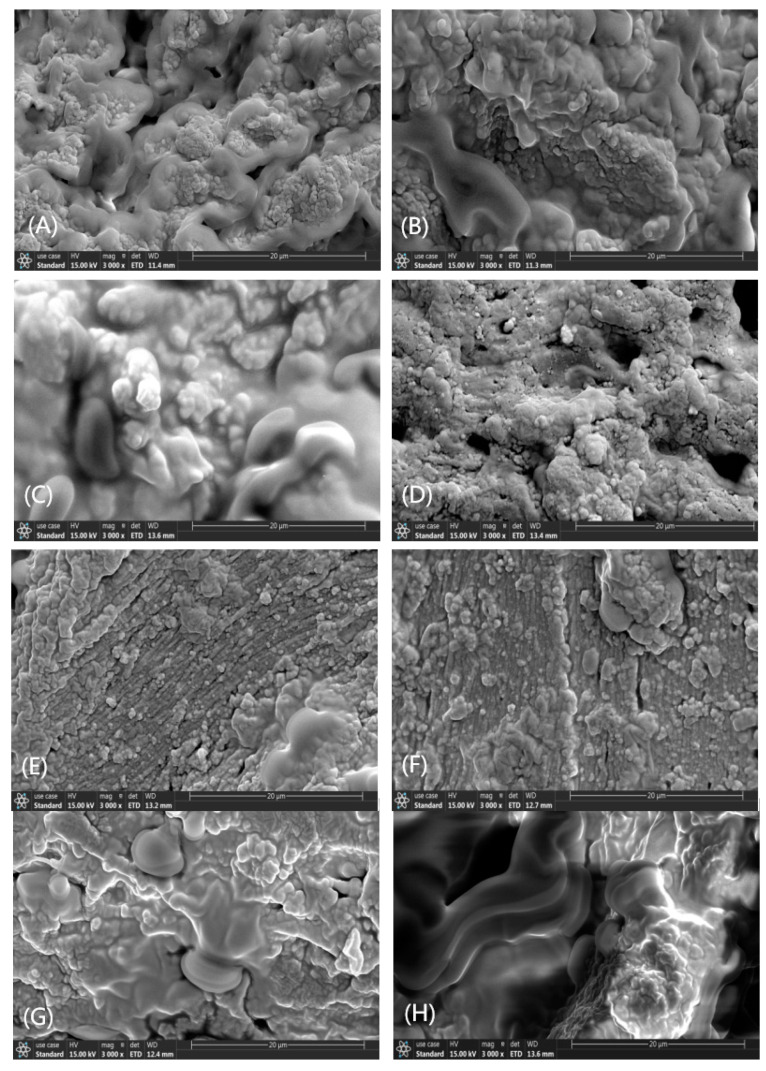
Scanning electron microscopy (SEM) images of pork meatballs formulated with different additives. The magnification was 3000×. (**A**) CON; (**B**) 0.5%κ-CAR; (**C**) 4%EWP; (**D**) 6%WG; (**E**) 5%SPI; (**F**) κ-CAR and EWP; (**G**) κ-CAR and WG; (**H**) κ-CAR and SPI.

The samples were examined at a magnification of ×3000 to examine the effect of exogenous additives on the microstructure of minced pork. The SEM images presented in Figure 3 highlighted the microstructures of minced pork samples amended with κ-CAR, EWP, WG, and SPI. In these images, the gel structure of the control minced pork appears sparse and disorganized, with large pores and an irregular shape, which explains the poor WHC and cooking rates associated with minced pork lacking exogenous additives. Minced pork containing κ-CAR, EWP, WG, and SPI exhibits a more homogeneous structure with no holes, which shows that additives can improve the gel network structure of the minced pork, effectively retaining moisture and thereby decreasing the cooking loss. The gel network of minced pork supplemented with κ-CAR/EWP and κ-CAR/WG was particularly compact and orderly, characterized by tiny pores. Specially, minced pork containing κ-CAR/WG forms larger spherical gel structures. Similar findings were reported in studies by Eyiler Yilmaz et al. and Kim et al. [41,44]. This enhanced gel structure is associated with a rise in disulfide bonds, affecting the textural properties. For the κ-CAR/SPI group, the gel matrix presents a coarse surface and irregular distribution, potentially attributable to the disruption in protein configurations within the minced pork induced by the excessive water absorption by the additives. These resonated with the aforementioned changes in the secondary structure of the proteins.

## 3. Conclusions

The moderate addition of κ-CAR and non-meat proteins demonstrated efficacy in elevating the WHC and textural properties of minced pork. It was observed that the augmentation of protein intermolecular hydrophobic interactions and the formation of disulfide bonds significantly enhanced the textural properties of minced pork. Furthermore, the transformed from α-helix to β-sheet created a more ordered gel network structure of minced pork. Thermal stability and SEM results revealed that the action of κ-CAR and non-meat proteins led to superior structural stability and an increased density within the minced pork gel network. As highlighted previously, the addition of κ-CAR and non-meat proteins can improve the gel characteristics and protein structure of minced pork, with the κ-CAR/WG group emerging as the most effective. Our study lays a foundational theoretical basis for improving the quality of minced pork. Further investigations should delve into the influence of κ-CAR and proteins on the sensory properties of minced pork. Additionally, it will be crucial to explore the effect of the pre-treatment of κ-CAR and proteins on the gel of minced pork.

## 4. Materials and Methods

### 4.1. Materials

Fresh pork hind legs and pork backfat were purchased from Yibin Daily Shopping Supermarket (Sichuan, China), divided into pieces of the same size, and stored in the refrigerator at −18 °C. κ-CAR, EWP, WG, and SPI used in this research were food-grade and purchased from Henan Wanbang Industrial Co., Ltd., Shangqiu, China. Sodium chloride (NaCl), urea, β-mercaptoethanol, trichloroacetic acid, sodium hydroxide (NaOH), and phosphoric acid were purchased from Chron Chemicals (Chengdu, China). All reagents were of analytical grade.

### 4.2. Preparation of Minced Pork

Basic formula: pork (fat to lean ratio 2:8), salt 2 g/100 g, ice water 2 g/100 g, κ-CAR and three proteins, all by meat weight (Table 4). Tendon and fascia were removed from the pork legs and backfat, and cut into small pieces. The meat and fat were minced separately with a 6 mm sieve (TK-12, Zhejiang Yingxiao Industry and Trade Co., Ltd., Jinhua, China). The minced pork was mixed with 2 g/100 g salt and 1/3 ice water for 3 min, fat and 1/3 ice water for 3 min, and then κ-CAR, protein, and 1/3 ice water for 3 min. The recipe was produced using three independent batches of minced pork at 0.5 kg per batch (50 g minced pork per group), and heated in an 80 °C water bath for 30 min (HWS-12, Shanghai Qixin Scientific Instruments Co., Ltd., Shanghai, China). All minced pork formulations were cooled, wrapped in cling film, and placed in a refrigerator at 4 °C overnight for the determination of relevant indicators.

### 4.3. Cooking Loss (CL)

A total of 2 g minced pork was individually were stuffed into a 50 mL centrifuge tube, followed by cooking at 80 °C for 10 min using a water bath. The samples were removed and quickly cooled to room temperature, and then weighed after drying the surface of the minced pork with absorbent paper.

CL (%) = W_2_/(W_1_ − W_0_) × 100 (1)

where W_0_ is the mass of the centrifuge tube (g), W_1_ is the sample mass before cooking (g), and W_2_ is the sample mass after cooking (g).

### 4.4. Water-Holding Capacity (WHC)

WHC was determined using the method of Mi et al. [45] with minor modifications. A total of 3 g minced pork was wrapped with filter paper and placed in a centrifuge tube, and then centrifuged at 3000× *g* for 10 min. The WHC of the gel was calculated according to the following equation.

WHC (%) = M_2_/M_1_ × 100 (2)

where M_1_ is the sample mass before centrifugation (g), and M_2_ is the sample mass after centrifugation (g).

### 4.5. Color

Minced pork samples were cut into 10 mm × 10 mm cylinders. The L* (lightness), a* (redness or greenness), and b* (yellowness or blueness) values of minced pork were determined using a standard white calibration and D65 light source (UltraScan VIS, HunterLab, Reton, VA, USA). The color was measured on the two round bottom and side surfaces of each sample to obtain an average value.

### 4.6. Texture Profile Analysis (TPA)

TPA parameters, namely, hardness, cohesiveness, springiness, and chewiness were determined using a texture analyzer (Stable Micro Systems), with a slight modification of the methodology described previously by Li et al. [46]. The samples were cut into cylindrical cores (20 mm length and 20 mm diameter). The samples compressed 5 s at 50% of their original height with a P/36R probe at a pre-test speed of 2.0 mm/s, a test speed of 2.0 mm/s, and a post-test speed of 5.0 mm/s. The mean value of the three readings is reported for each attribute.

### 4.7. Secondary Structure via Raman Spectroscopy

Secondary structures were conducted using Raman spectroscopy (INVIA, Renishaw, London, UK). Minced pork was evenly placed on a slide and put on a carrier table to determine its spectrum with parameters referenced in the method of Xu et al. [47]. The spectra were recorded in the range of 100 to 3500 cm^−1^. Each spectrum was under the following conditions: 100 mW laser power, 3 scans, 60 s exposure time, 2 cm^−1^ resolution, and a sampling speed of 120 cm^−1^. Protein secondary structures were determined as a percentage of α-helix (%), β-sheet (%), β-turn (%), and random coil (%) using Alix’s method [48]. Each sample was evaluated in triplicate.

### 4.8. Thermal Stability Analysis

Thermal stability including the denaturation temperature (°C) and enthalpy value (J/g) of minced pork was assessed using a differential scanning calorimeter (DSC200F3, NETZSCH Scientific Instruments Trading, Weimar, Germany). Briefly, 15 mg samples were sealed in an aluminum dish, recorded, and placed in the sample cell with the empty crucible as a control. After initial equilibration at 20 °C for 10 min, the samples were heated continuously from 20 °C to 100 °C at a rate of 3 °C min^−1^. Each sample was tested in triplicate.

### 4.9. Determination of Intermolecular Forces

The intermolecular forces of minced pork gels were measured by the method of TAN et al. [49]. The samples were dissolved in four solutions in sequence: 0.6 mol/L NaCl solution (A), 0.6 mol/L NaCl + 1.5 mol/L urea (B), 0.6 mol/L NaCl + 8 mol/L urea (C), and 0.6 mol/L NaCl + 8 mol/L urea + 0.5 mol/L β-mercaptoethanol (D). A total of 1 g minced pork gel was chopped and homogenized (FJ200-SH, Shanghai Huxi Industrial Co., Ltd., Shanghai, China) with 10 mL A solution. The resulting precipitate was homogenized with 10 mL B solution, 10 mL C solution, and 10 mL D solution, in that order. After homogenization, the gel was left at 4 °C for 1 h and then centrifuged at 4 °C and 10,000× *g* for 25 min, and the resulting four supernatants were stored at 4 °C. The supernatant from the above steps was added to an equal volume of 20% TCA solution (trichloroacetic acid solution), mixed, and centrifuged for 15 min at 5000× *g*. The supernatant was poured off, and 1 mL NaOH (1 mol/L) solution was incorporated into the precipitate and stored at 4 °C. The protein content in fluids A, B, C, and D corresponds to the content of ionic bonds, hydrogen bonds, hydrophobic interactions, and disulfide bonds, respectively. Each sample was evaluated in triplicate.

### 4.10. Scanning Electron Microscopy (SEM)

Scanning electron microscopy (SEM) images were obtained using Álvarez et al.’s [22] method with slight modification. Each sample was diced into small pieces and immersed in 2.5% glutaraldehyde (phosphate buffer, pH 7.4) for more than 12 h at 4 °C, and then transferred into 0.1 M phosphate buffer for 10 min. Samples were dehydrated at serial concentrations of 30%, 50%, 70%, 80%, 90%, and 100% ethanol and acetone for 10 min, respectively. The samples were dried at 50 °C and then sputtered with gold in a sputter coater, observed, and photographed. Specimens were viewed using a scanning electron microscope (JSM-7500F, JEOL, Tokyo, Japan).

### 4.11. Statistical Analysis

One-way analysis of variance (ANOVA) followed by Duncan’s multiple range tests was performed (*p* < 0.05). Statistical analyses were conducted with the statistical program SPSS 26.0 (SPSS Inc., Chicago, IL, USA) for Windows. All graphs in this study were obtained using Origin 2018. All experiments were repeated 3 times and results were expressed as means ± standard error.

## Figures and Tables

**Table 1:** Cooking loss, WHC, and whiteness of minced pork formulated with different additives (mean ± standard error).

Treatment ^1^	Cooking Loss (%)	WHC ^2^ (%)	L*	a*	b*
Con	17.26 ± 0.22 ^a^	75.25 ± 0.14 ^g^	74.90 ± 0.06 ^a^	1.10 ± 0.03 ^d^	11.68 ± 0.09 ^b^
κ-CAR	16.73 ± 0.05 ^b^	77.95 ± 0.28 ^f^	74.61 ± 0.44 ^a^	1.60 ± 0.10 ^abc^	13.55 ± 0.20 ^a^
WG	10.92 ± 0.10 ^d^	78.77 ± 0.47 ^e^	74.07 ± 0.50 ^a^	1.91 ± 0.22 ^a^	13.10 ± 0.23 ^a^
EWP	10.62 ± 0.23 ^d^	79.95 ± 0.11 ^d^	75.03 ± 0.24 ^a^	1.62 ± 0.14 ^ab^	12.99 ± 0.73 ^a^
SPI	1.31 ± 0.01 ^e^	91.42 ± 0.20 ^a^	72.51 ± 0.35 ^b^	1.28 ± 0.16 ^bcd^	13.07 ± 0.44 ^a^
C-E	16.18 ± 0.30 ^c^	77.53 ± 0.22 ^f^	74.53 ± 0.05 ^a^	1.22 ± 0.05 ^cd^	12.53 ± 0.23 ^b^
C-W	10.98 ± 0.16 ^d^	81.91 ± 0.07 ^c^	74.50 ± 0.19 ^a^	1.40 ± 0.03 ^bcd^	12.52 ± 0.38 ^b^
C-S	0.98 ± 0.14 ^e^	87.37 ± 0.23 ^b^	74.53 ± 0.05 ^a^	1.22 ± 0.05 ^cd^	12.52 ± 0.23 ^b^

^1^ Con, control; κ-CAR (0.5 g/100 g κ-carrageenan); EWP (4 g/100 g egg white powder); WG (wheat gluten); SPI (5 g/100 g soy isolate protein); C-E (0.5 g/100 g κ-carrageenan and 4 g/100 g egg white powder); C-W (0.5 g/100 g κ-carrageenan and 6 g/100 g wheat gluten); C-S (0.5 g/100 g κ-carrageenan and 5 g/100 g soy isolate protein). ^2^ WHC, water holding capacity. ^a–f^ Different letters in the same column indicate significant differences (*p* < 0.05).

**Table 2:** Textural properties of pork meatballs formulated with different additives (mean ± standard error).

Treatment ^1^	Hardness (N)	Cohesiveness	Springiness (%)	Chewiness (N)
Con	20.61 ± 1.21 ^f^	0.38 ± 0.01 ^b^	0.82 ± 0.01 ^c^	6.35 ± 7.94 ^e^
κ-CAR	19.86 ± 0.89 ^g^	0.34 ± 0.01 ^c^	0.74 ± 0.01 ^d^	4.90 ± 0.47 ^f^
EWP	25.82 ± 0.91 ^c^	0.41 ± 0.01 ^a^	0.83 ± 0.01 ^c^	8.79 ± 0.58 ^c^
WG	38.91 ± 0.80 ^a^	0.43 ± 0.01 ^a^	0.88 ± 0.01 ^a^	14.73 ± 0.41 ^a^
SPI	22.56 ± 0.23 ^d^	0.31 ± 0.01 ^d^	0.66 ± 0.01 ^e^	4.56 ± 0.03 ^g^
C-E	21.61 ± 0.29 ^e^	0.39 ± 0.01 ^b^	0.82 ± 0.01 ^c^	6.86 ± 0.27 ^d^
C-W	27.93 ± 0.38 ^b^	0.42 ± 0.01 ^a^	0.85 ± 0.01 ^b^	9.83 ± 0.36 ^b^
C-S	20.59 ± 0.24 ^f^	0.24 ± 0.01 ^e^	0.57 ± 0.02 ^f^	2.82 ± 0.15 ^h^

^1^ Con, control; κ-CAR (0.5 g/100 g κ-carrageenan); EWP (4 g/100 g egg white powder); WG (wheat gluten); SPI (5 g/100 g soy isolate protein); C-E (0.5 g/100 g κ-carrageenan and 4 g/100 g egg white powder); C-W (0.5 g/100 g κ-carrageenan and 6 g/100 g wheat gluten); C-S (0.5 g/100 g κ-carrageenan and 5 g/100 g soy isolate protein). ^a–h^ Different letters in the same column indicate significant differences (*p* < 0.05).

**Table 3:** The denaturation temperature (Td) and enthalpy (∆H) of pork meatball protein formulated with different additives (mean ± standard error).

Treatment ^1^	Td (°C)	∆H (J/g)
Con	66.70 ± 0.12 ^a^	0.33 ± 0.04 ^g^
κ-CAR	63.50 ± 0.06 ^d^	0.39 ± 0.02 ^f^
EWP	65.00 ± 0.17 ^b^	0.45 ± 0.00 ^e^
WG	63.40 ± 0.23 ^d^	0.68 ± 0.02 ^b^
SPI	64.00 ± 0.29 ^cd^	0.57 ± 0.02 ^c^
C-E	65.43 ± 0.09 ^b^	0.51 ± 0.01 ^d^
C-W	64.00 ± 0.23 ^b^	0.75 ± 0.01 ^a^
C-S	64.30 ± 0.25 ^cd^	0.51 ± 0.03 ^d^

^1^ Con, control; κ-CAR (0.5 g/100 g κ-carrageenan); EWP (4 g/100 g egg white powder); WG (wheat gluten); SPI (5 g/100 g soy isolate protein); C-E (0.5 g/100 g κ-carrageenan and 4 g/100 g egg white powder); C-W (0.5 g/100 g κ-carrageenan and 6 g/100 g wheat gluten); C-S (0.5 g/100 g κ-carrageenan and 5 g/100 g soy isolate protein). ^a–g^ Different letters in the same column indicate significant differences (*p* < 0.05).

**Table 4:** Quantity of ingredients used in pork balls formulations.

Treatment ^1^	Amount of Each Substance Added to Minced Meat ^2^							
	Meat	Backfat	κ-CAR	EWP	WG	SPI	Salt	Ice Water
Con	80	20	-	-	-	-	2	20
κ-CAR	80	20	0.5	-	-	-	2	20
WG	80	20	-	4	-	-	2	20
EWP	80	20	-	-	6	-	2	20
SPI	80	20	-	-	-	5	2	20
C-E	80	20	0.5	4	-	-	2	20
C-W	80	20	0.5	-	6	-	2	20
C-S	80	20	0.5	-	-	5	2	20

^1^ Con, control; κ-CAR (0.5 g/100 g κ-carrageenan); EWP (4 g/100 g egg white powder); WG (wheat gluten); SPI (5 g/100 g soy isolate protein); C-E (0.5 g/100 g κ-carrageenan and 4 g/100 g egg white powder); C-W (0.5 g/100 g κ-carrageenan and 6 g/100 g wheat gluten); C-S (0.5 g/100 g κ-carrageenan and 5 g/100 g soy isolate protein). ^2^ κ-CAR, EWP, WG and SPI, salt, and ice water all by weight of meat.

## Data Availability

The data presented in this study are openly available in the article.
